# Households as Foci for Dengue Transmission in Highly Urban Vietnam

**DOI:** 10.1371/journal.pntd.0003528

**Published:** 2015-02-13

**Authors:** Katherine L. Anders, Le Hong Nga, Nguyen Thi Van Thuy, Tran Van Ngoc, Cao Thi Tam, Luong Thi Hue Tai, Nguyen Thanh Truong, Huynh Thi Le Duyen, Vu Tuan Trung, Duong Thi Hue Kien, Marcel Wolbers, Bridget Wills, Nguyen Van Vinh Chau, Nguyen Dac Tho, Cameron P. Simmons

**Affiliations:** 1 Oxford University Clinical Research Unit—Wellcome Trust Major Overseas Programme, Ho Chi Minh City, Vietnam; 2 Centre for Tropical Medicine, University of Oxford, Oxford, United Kingdom; 3 Department of Epidemiology and Preventive Medicine, Monash University, Melbourne, Australia; 4 Preventive Medicine Centre, Ho Chi Minh City, Vietnam; 5 Hospital for Tropical Diseases, Ho Chi Minh City, Vietnam; 6 Department of Microbiology and Immunology, University of Melbourne, Melbourne, Australia; Pediatric Dengue Vaccine Initiative, UNITED STATES

## Abstract

**Background:**

Dengue control programs commonly employ reactive insecticide spraying around houses of reported cases, with the assumption that most dengue virus (DENV) transmission occurs in the home. Focal household transmission has been demonstrated in rural settings, but it is unclear whether this holds true in dense and mobile urban populations. We conducted a prospective study of dengue clustering around households in highly urban Ho Chi Minh City, Vietnam.

**Methods:**

We enrolled 71 index cases with suspected dengue (subsequently classified as 52 dengue cases and 19 non-dengue controls); each initiated the enrollment of a cluster of 25–35 household members and neighbors who were followed up over 14 days. Incident DENV infections in cluster participants were identified by RT-PCR, NS1-ELISA, and/or DENV-IgM/-IgG seroconversion, and recent infections by DENV-IgM positivity at baseline.

**Principal Findings/Conclusions:**

There was no excess risk of DENV infection within dengue case clusters during the two-week follow-up, compared to control clusters, but the prevalence of recent DENV infection at baseline was two-fold higher in case clusters than controls (OR 2.3, 95%CI 1.0–5.1, p = 0.05). Prevalence of DENV infection in *Aedes aegypti* was similar in case and control houses, and low overall (1%). Our findings are broadly consistent with household clustering of dengue risk, but indicate that any clustering is at a short temporal scale rather than sustained chains of localized transmission. This suggests that reactive perifocal insecticide spraying may have a limited impact in this setting.

## Author Summary

Dengue is a viral disease transmitted between humans by *Aedes aegypti* mosquitoes. It is an increasing health problem in tropical regions, with no vaccine or drug available. The main approach used for dengue prevention and control is reducing mosquito numbers, which is commonly done by spraying insecticide around the houses of reported dengue cases and their neighbors. This assumes that mosquito populations around the home contribute to onwards transmission of dengue to other people living near the identified case, and that by reducing these populations further infections can be prevented. A previous study in rural villages in Thailand showed that dengue infections did cluster near the house of a case, but it is not clear whether this would also be the case in a densely-populated urban setting like Ho Chi Minh City. In this study, we show that people living close to a dengue case in HCMC were in fact no more likely to be infected with dengue over a two week period than controls who did not live near a dengue case, although they were two times more likely to have evidence of a recent dengue infection at enrollment. This suggests that dengue risk might cluster around households in this setting, but that responsive insecticide spraying around the homes of reported cases is unlikely to prevent many additional infections.

## Introduction

Dengue represents a large and growing public health problem throughout the tropical and sub-tropical world, with nearly 100 million clinical cases estimated to occur annually [1]. Dengue results from infection with one of four serotypes of dengue virus (DENV-1–4), transmitted between humans primarily by *Aedes aegypti* mosquitoes. *Ae*. *aegypti* are highly efficient vectors due to their preference for human blood meals, and for breeding, feeding and resting in and around domestic areas [2]. Despite considerable research, the most advanced dengue vaccine candidate has only intermediate efficacy [3], making control of the mosquito vector an ongoing feature of dengue prevention and epidemic response for the foreseeable future.

Unfortunately vector control efforts have failed to curb dengue’s rise over the past 50 years, during which time both the case numbers and geographic range have increased dramatically. With the notable exceptions of the *Ae*. *aegypti* eradication program in the Americas [4] and sustained periods of vector suppression in Singapore [5] and Cuba [6], the operational focus of *Ae*. *aegypti* control in endemic countries has been on reducing vector abundance to achieve a concomitant reduction in dengue transmission, rather than total elimination. However the threshold below which vector density must fall to prevent dengue transmission is unknown, and the experience of Singapore demonstrates that transmission is possible even at very low vector densities [5]. The resources required to achieve broad and sustained suppression of *Ae*. *aegypti* exceed the national budgets of dengue control programs in many endemic countries, and therefore a targeted reactive approach is commonly used based on perifocal insecticide spraying in and around the houses of reported dengue cases and their neighbors. Despite the frequency with which this approach is used, there is little published data evaluating its efficacy in preventing dengue transmission.

Dengue incidence is highly heterogeneous is both space and time. Spatial clustering of dengue has been described both at an intermediate scale [7,8], and at fine spatial scales in several studies focused on vectors, DENV infections and dengue cases. Adult and immature vector indices are commonly highly clustered in space and time at the household level [9,10] and *Ae aegypti* are unlikely to fly further than 100m within their life span if suitable human blood meals and ecological conditions are available close by [11]. This spatial distribution of entomological risk is consistent with observations of fine scale clustering of dengue. A study from rural Thailand reported remarkably focal DENV infection risk in children and mosquitoes at the fine scale of 100m and 2 weeks [12,13]. This supports the home as a focus for dengue transmission in that setting and therefore also as a target for control. However it is not clear to what degree this focal pattern holds true in a highly urban dense population, which may be more mobile and mixed. Two studies using a similar index-cluster design were conducted in urban settings in Indonesia [14] and Nicaragua [15], but included either no or very few control clusters, respectively, so could not draw conclusions on spatial clustering of DENV infections. A study in Bangkok of a retrospective series of hospitalized dengue cases with known serotype found that homotypic cases were significantly clustered at a scale of approximately 1km and ≤4 months [16], suggesting that local population immunity shapes subsequent disease distribution at a fine spatial scale. However a prospective study of fine-scale dengue clustering in a large urban setting has not been published previously.

This study aimed to investigate whether households are important foci of DENV infection in urban centers, or whether the distribution of dengue risk in a dense highly urban population is more generalized. This has implications for the rational implementation of vector control interventions in dengue-endemic resource-limited settings. Here we report the results of a prospective study of dengue clustering around households in highly urban Ho Chi Minh City, Vietnam.

## Methods

### Study setting, design and enrollment

Our study was conducted between October 2010 and January 2013 in Ho Chi Minh City (HCMC)—the largest city in Vietnam. Participants came from seven contiguous districts in central HCMC comprising 1.6 million inhabitants within 129 km^2^ (population density 12,400/km^2^). The Hospital for Tropical Diseases (HTD) is the referral hospital for infectious diseases in southern Vietnam, located in central HCMC.

Index participants were non-consecutive patients with a clinical suspicion of dengue, admitted to HTD within 96 hours of illness and residing within the specified catchment area. An additional 6 index participants, who met the same criteria but were ambulatory patients, were enrolled at a private clinic. All ages were eligible, but patients in intensive care were excluded because few would have illness ≤96 hours. Up to five participants were enrolled within each two-week period, with numbers constrained by the time needed for enrollment and follow-up of each cluster. Index participants were later classified as either index cases or controls based on laboratory diagnostics, as described below; this strategy for enrolment of index cases and controls was used so that cluster enrolment could begin quickly without waiting for diagnostic results, and so that field staff were blinded to the index case or control status throughout follow-up.

Within 2 days of index enrollment, field staff visited the index participant’s home and enrolled a cluster of 25–30 household members and close neighbors living within 100m of the index house. All ages were eligible, but preference was given to children <15 years as the age group at (presumed) highest risk of DENV infection. Cluster participants were visited again 7 and 14 days after enrollment.

### Data and sample collection

Venous blood was collected from index participants at enrollment and hospital discharge. Data was recorded on demographics, household composition and mobility. Laboratory diagnostics were done retrospectively by batch assay, such that field staff, laboratory staff and participants were blinded to the index’s case or control status throughout data collection and follow-up.

Data on cluster participants’ demographics, usual daily activities, and any febrile illness in the week prior to each study visit were recorded. A capillary blood sample was collected at each of the 3 study visits, with 4 drops of blood (~20ul each) collected as separate spots onto filter paper (Whatman 3MM, Sigma-Aldrich). Dried blood spots have previously been show to have high sensitivity for detection of DENV IgM, IgG, NS1 and viral RNA, compared with plasma [17]. Blood spots were dried and stored with desiccant at 4°C until testing. Geographic coordinates of participants’ houses were recorded. Field staff used backpack aspirators to collect adult mosquitoes inside index houses for 15 minutes at each of 3 study visits. Mosquitoes were sorted visually by species and sex and stored at -80°C until testing.

Cluster participants were asked to present to a study nurse at HTD outpatient department at any time during the follow-up period in the event of febrile illness. A 2ml venous blood sample was collected from any participant with self-reported or documented fever, either at a study visit or presenting to the study nurse.

### Laboratory diagnostics

Plasma samples from index participants were tested by IgM/IgG antibody capture ELISA using DENV/Japanese encephalitis virus antigens and mAb reagents from Venture Technologies (Sarawak, Malaysia) as described previously [18]. Enrollment samples were tested for DENV viremia using a validated, quantitative RT-PCR assay as described previously [19], and for DENV NS1 antigen using a commercial ELISA assay (Biorad Platelia) according to manufacturers’ instructions. Plasma samples collected from cluster participants reporting fever were also tested for DENV infection as above.

Individual dried blood spot (DBS) samples from cluster participants were eluted into 400ul PBS/Tween-20 (0.05%) as described previously [17], and tested by IgM/IgG capture ELISA. Where serology results indicated IgM and/or IgG seroconversion, additional DBS samples from study visits immediately pre- and post-conversion were eluted into 1) 500ul RPMI medium and tested by NS1 Platelia ELISA, and 2) 560ul viral lysis buffer (QIAamp Viral RNA Mini kit, Qiagen, Vanecia, CA) for RNA extraction and quantitative RT-PCR.

Female *Aedes aegypti* collected from index households were individually homogenized with 1mm zirconia/silica beads for 15 minutes at 30 HZ using a TissueLyser II (Qiagen). Pools of ten homogenates were tested for DENV infection by quantitative RT-PCR; positive pools were then assayed individually.

### Case definitions and endpoints

An index participant was classified as having laboratory-confirmed dengue if they seroconverted by IgM and/or IgG capture ELISA, or had a positive result in DENV RT-PCR or NS1 ELISA. Index participants negative by all assays were classified as ‘other febrile illness’ controls. Index participants cases with negative results in RT-PCR and NS1 ELISA and lacking a second plasma sample were deemed unclassifiable, as seroconversion could not be excluded.

The primary endpoint was incident DENV infection among cluster participants, defined by IgM/IgG seroconversion between any two samples, or a positive RT-PCR or NS1 result. Infections were subsequently classified as inapparent, or symptomatic based on a self-reported or documented fever during the follow-up period. The secondary endpoint was recent DENV infection among cluster participants at baseline, defined by a positive IgM result.

### Statistical considerations

Data were analyzed using Stata version 11. Mapping used ArcMap version 10.1. Comparisons of continuous variables with non-normal distributions were made between groups using the Wilcoxon rank-sum test. We analyzed the relative risk of recent and incident DENV infections in dengue positive versus negative clusters using mixed effects logistic regression models with a fixed effect for the index case infection status and a random effect to model between-cluster heterogeneity. Unadjusted models and models adjusted for seasonality (quarter of enrollment) and participants’ age were performed. Additional adjustment for insecticide use was done *post hoc*, after observing a large difference in use between positive and negative clusters. A secondary analysis used a mixed effects logistic regression model to explore determinants of dengue transmission risk within case clusters only. Variables of interest defined *a priori* included cluster-level variables (age/sex of index case, enrollment viremia, serotype, baseline *Ae*. *aegypti* abundance, insecticide spraying at baseline, and average daytime hours the index case spends at home), and individual-level variables (age/sex of cluster participant, whether household member or neighbor, and average daytime hours spent at home).

### Ethical considerations

The study protocol was approved by the ethical committees of 1) HTD, HCMC (approval reference CS/ND/10/16) 2) Centre for Preventive Medicine, HCMC, 3) Oxford University Tropical Ethics Committee (OXTREC; approval ref. 21–10), and 4) Monash University Human Research Ethics Committee (MUHREC; approval ref. 2010001725). Written informed consent was obtained from all participants prior to enrollment, or from parents/guardians of participants <16 years. Patients 16 years and over are considered adults within the Vietnamese health care system, and have the autonomy to give independent consent for clinical research when approved by an Ethics Committee.

## Results

### Characteristics and classification of index cases

**Fig 1:**
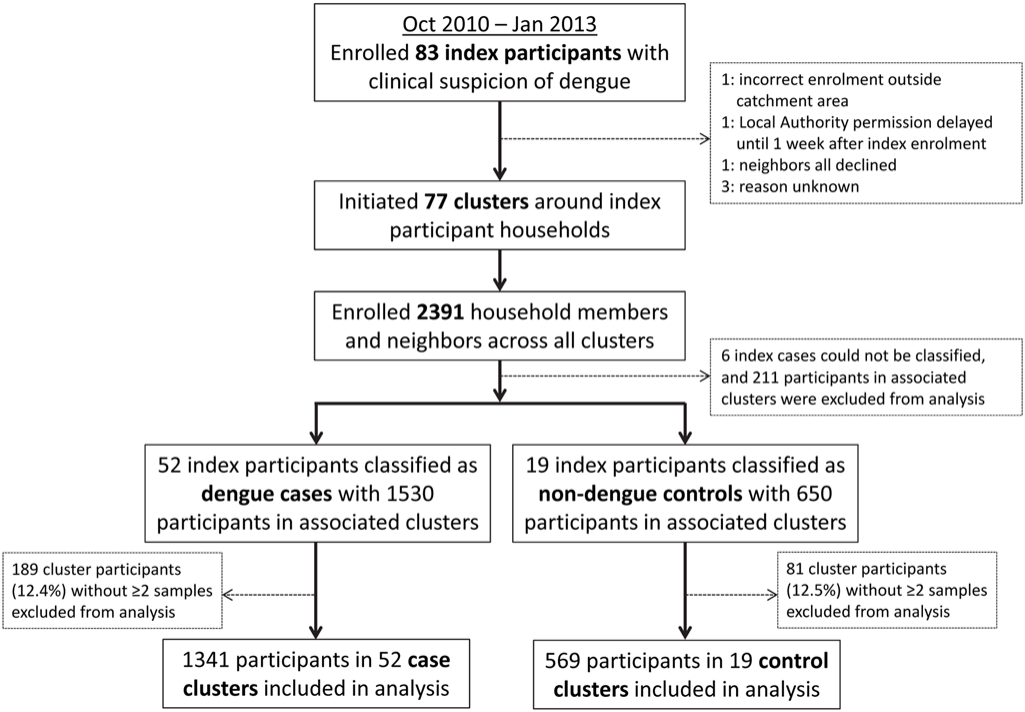
Summary of study design and enrollment.

**Fig 2:**
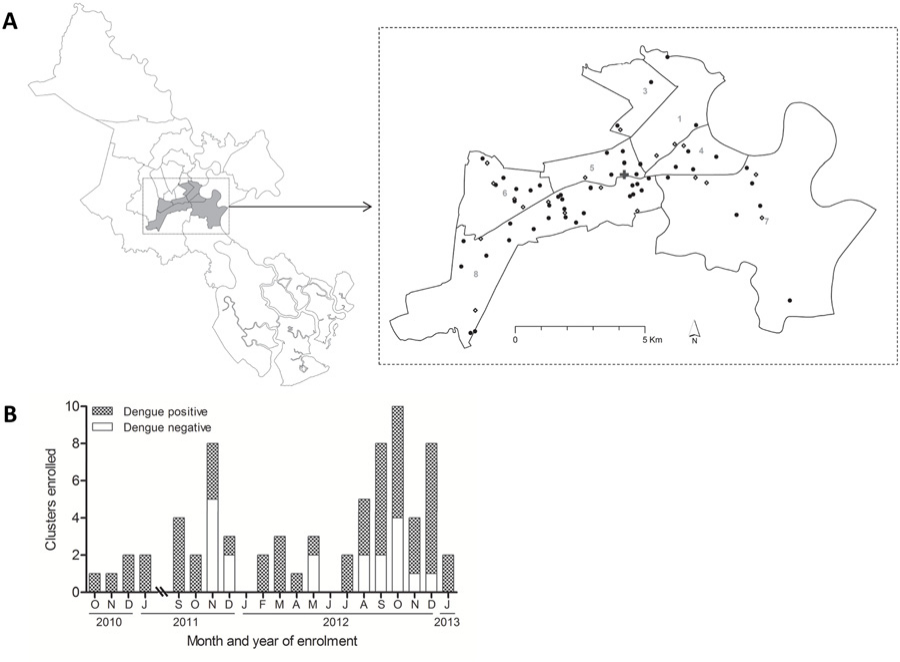
Spatial and temporal distribution of cluster investigations. A: Map of Ho Chi Minh City, with inset showing the spatial distribution of dengue positive clusters (filled circles) and negative clusters (open diamonds) within 7 central districts (district numbers are shown). B: Number of dengue positive and negative cluster investigations, by month of enrollment.

Among 83 index participants enrolled, a cluster investigation was initiated for 77. Reasons for a failure to initiate a cluster are detailed in Fig. 1. Subsequent laboratory investigations classified 52 as laboratory–confirmed dengue cases and 19 as other febrile illness controls. In a further 6 index participants, DENV infection could not be confirmed or excluded; these were deemed indeterminate and their associated clusters were excluded from analysis (S1 Table). DENV-1 and DENV-2 each accounted for one quarter of DENV-positive cases (Table 1). Almost 40% of dengue cases and 26% of controls were children <15 years. The enrollment of cases and controls was similarly distributed in space and time (Fig. 2), with two-thirds of all index participants enrolled in 2012.

**Table 1:** Characteristics of index cases.

	Dengue cases		Non-dengue controls	
Index cases enrolled	55		21	
With a linked cluster	52		19	
Year enrolled				
2010	4	(7.7)	0	(0)
2011	12	(23.1)	7	(36.8)
2012	34	(65.4)	12	(63.2)
2013	2	(3.9)	0	(0)
Female, n (%)	33	(63.5)	15	(79.0)
Age group, n (%)				
<5	1	(1.9)	1	(5.3)
5–<10	7	(13.5)	0	(0)
10–<15	12	(23.1)	4	(21.1)
15–<35	28	(53.9)	4	(21.1)
35–<55	3	(5.8)	10	(52.6)
55+	1	(1.9)	0	(0)
Day of illness, n (%)				
1–2	12	(23.1)	4	(21.1)
3–4	38	(73.1)	15	(79.0)
5	2	(3.9)	0	(0)
Hours at home 6am–6pm, mean (sd)	6.9	(3.4)	8.4	(3.6)
Travels outside own district ≥1/ week, n (%)	15	(28.9)	3	(16.7)
NS1 positive, n (%)	37	(71.2)	–	–
Serotype, n (%)				
DENV-1	13	(25.0)	–	–
DENV-2	12	(23.1)	–	–
DENV-3	4	(7.7)	–	–
DENV-4	10	(19.2)	–	–
PCR-negative	13	(25.0)	–	–

### Characteristics of cluster participants

A total of 2391 household members and neighbors were enrolled across the 77 cluster investigations. Of these, 1341 participants in 52 case clusters and 569 participants in 19 control clusters had at least two samples collected and were included in the analysis (Fig. 1). Case and control clusters were comparable with respect to age, sex, and other variables (Table 2).

**Table 2:** Cluster composition and characteristics of cluster participants.

	Dengue positive clusters		Dengue negative clusters	
Number of clusters	52		19		
Participants enrolled,total (household: neighbors)	1341	(85:1256)	569	(33:536)
Cluster size, median (range)	30	(18–41)	33	(29–41)
Female, n (%)	808	(62.0%)	335	(61.1%)
Median age in years (range)	38.3	(0–90)	38.8	(0–84)
Age group, n (%)				
<5	125	(9.4%)	52	(9.1%)
5–<15	166	(12.4%)	79	(13.9%)
15–<35	309	(23.2%)	136	(23.9%)
35–<55	448	(33.6%)	167	(29.4%)
55+	287	(21.5%)	135	(23.7%)
Hours at home 6am–6pm, mean (sd)	7.7	(2.4)	7.8	(2.4)
Travels outside own district ≥1/week, n (%)	149	(11.2%)	56	(9.8%)
Insecticide sprayed at home in 3 weeks prior to enrollment	35	(2.7%)	89	(16.3%)
Insecticide sprayed at home during two-week follow-up period	805	(60.8%)	410	(73.0%)
Participants with blood sample collected at:				
Visit 1	1341		569	
Visit 2*	1146	(85.5%)	496	(87.2%)
Visit 3*	1253	(93.4%)	534	(93.8%)

*Denominator for percentages is the number of participants with an enrollment sample collected at Visit 1.

### DENV infection prevalence in *Aedes aegypti*

In total 790 female *Aedes aegypti* were collected across 3 collections at each of the 71 index households (Table 3). Vector density varied substantially, with the median of the sum of three collections being seven female *Ae aegypti* per household (range 0–57). Although the median vector density was comparable between case and control households, the distribution was positively skewed with a significantly higher occurrence of very high densities in case households than in controls (Wilcoxon rank-sum z = -6.25, p<0.0001). Eight individual *Ae aegypti* were DENV-PCR positive (1% of total); there was no difference in the frequency of DENV-positive mosquitoes or the proportion of houses with an infected mosquito between case and control households (Fisher’s exact p = 1.0).

**Table 3:** Mosquito collections and DENV infections in *Aedes aegypti*.

	Positive clusters	Negative clusters	Total
Clusters enrolled	52	19	71
Mosquito surveys	156	57	213
Female Ae. aegypti	644	146	790
Median Ae. aegypti (range) per cluster	8 (0–57)	6 (0–18)	7 (0–57)
Engorged Ae. aegypti (%)	264 (41.0%)	59 (40.4%)	323 (40.9%)
Median engorged (range) per cluster	2 (0–33)	3 (0–7)	3 (0–33)
DENV-positive clusters, n (% of clusters)	5 (9.6%)	2 (10.5%)	7 (9.9%)
DENV-positive surveys, n (% of surveys)	6 (3.8%)	2 (3.5%)	8 (3.8%)
DENV-positive aegypti, n (% of females)	6 (0.9%)	2 (1.4%)	8 (1.0%)

At enrollment, 3% of participants in case clusters and 16% in control clusters reported insecticide spraying at their home by public health authorities during the previous 3 weeks (Table 2); this increased to 61% and 73%, respectively, during the two-week follow-up period.

### DENV infections in cluster participants

A total of 113 incident DENV infections were detected among cluster participants; 82 among 1341 participants in dengue positive clusters (6.1%) and 31/569 in negative clusters (5.4%; Table 4). Surprisingly, the incidence of DENV infection was similar across age strata (S2 Table). Nine (8.0%) infections were associated with self-reported or documented febrile illness. Seven of nine symptomatic cases were in dengue positive clusters, and viremia was detectable in 6/9 cases: 3 were DENV-1, 2 DENV-2 and 1 DENV-4. In four cases the serotype matched that of the corresponding index case. In one DENV-2 case the index case had DENV-1, and one DENV-1 case was in a dengue negative cluster.

**Table 4:** DENV infections among cluster participants.

	Positive clusters		Negative clusters		Odds Ratio (95%CI)*	
					Unadjusted	Adjusted
Cluster participants included in analysis	1341		569			
Incident DENV infection during 2-week follow up	82	6.1%	31	5.4%	1.1 (0.6–2.0)	1.2 (0.6–2.4)
Symptomatic	7	0.52%	2	0.35%	1.4 (0.2–9.4)	–
Inapparent	75	5.6%	29	5.1%	–	–
Recent DENV infection at baseline	159	11.9%	29	5.1%	**2.3 (1.2–4.7)^1^**	**2.3 (1.0–5.2)^2^**
No DENV infection	875	65.3%	411	72.2%	–	–
Unclassifiable	225	16.8%	98	17.2%	–	–

*From logistic regression with random effect for clustering; adjusted model included age of index and cluster participant, and season of enrollment. Adjusted model could not be fitted for symptomatic cases due to small number of events.

^1^ p = 0.018

^2^ p = 0.05

**Fig 3:**
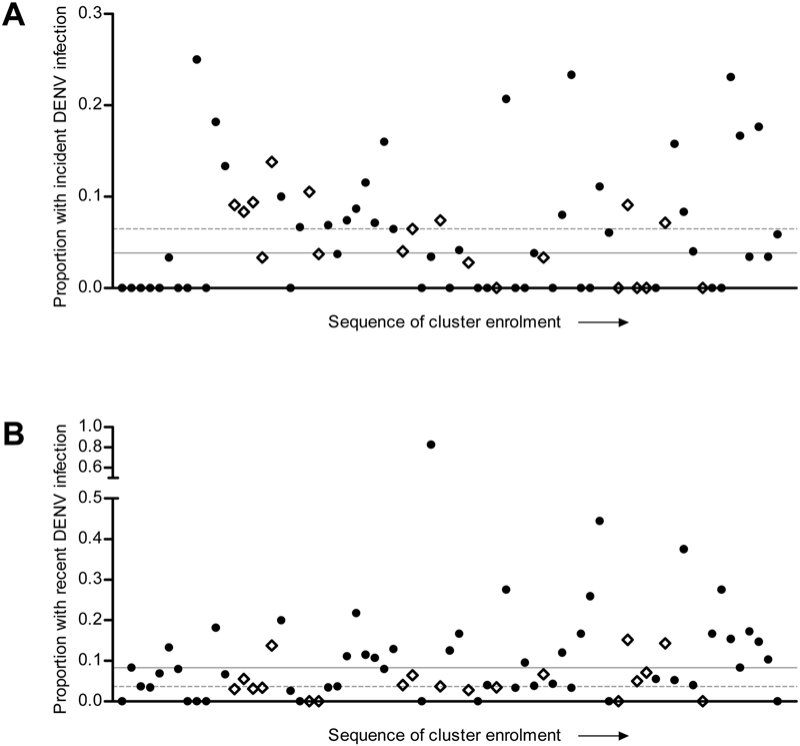
Incidence of DENV infection and prevalence of recent infection at baseline, by cluster. Each point represents the proportion of participants with incident DENV infection (A) or recent DENV infection at baseline (B) for individual clusters, in chronological sequence of enrollment. Dengue positive clusters are represented by filled circles and dengue negative clusters by open diamonds. The median cluster-level incidence (A) or prevalence (B) is shown for positive clusters by a solid line, and for negative clusters by a dashed line.

A further 159 (11.9%) participants in case clusters had detectable DENV-reactive IgM at baseline, suggestive of a recent infection, compared with 29 (5.1%) in control clusters. Fig. 3 shows DENV infection incidence and prevalence of recent infection for individual clusters. Full details of laboratory diagnostic results for cluster participants are shown in S1 Fig.

### Clustering of DENV infection risk

The results of mixed effects regression modeling confirmed no evidence of an association between the infection status of the index participant and the incidence of new DENV infections (total or symptomatic) among their household members and neighbors during the two-week follow-up period (Table 4). These results were unchanged by adjustment for insecticide use. In contrast, participants in case clusters were significantly more likely than those in control clusters to have detectable DENV-reactive IgM at baseline, both in univariate analysis and after adjustment for participant age and the season of enrollment (OR 2.3, 95%CI 1.0–5.1, p = 0.05). After additional adjustment for insecticide use in the three weeks prior to enrollment this association persisted but with wider confidence intervals (OR 2.2, 95%CI 0.9–5.1, p = 0.08).

### Risk factors for focal DENV transmission

Regression models were used to determine whether specified *a priori* risk factors were associated with DENV infection risk within dengue positive clusters. Only female *Ae*. *aegypti* density at baseline was associated in univariate analyses with the risk of incident DENV infection among participants in dengue positive clusters (OR 1.1 for each additional mosquito, 95%CI 1.0–1.2, p = 0.03). Unexpectedly, the average daytime hours participants spent at home was significantly negatively associated with the probability of them being IgM positive at baseline (OR 0.88, 95%CI 0.81–0.96, p = 0.006). Participants resident in an index case house had no greater risk of incident or recent DENV infection than participants living in neighboring houses.

## Discussion

This study of fine-scale clustering of dengue in urban HCMC found no excess risk of DENV infection among household members and neighbors of confirmed dengue cases, compared with equivalent contacts of controls, during the two-week period following enrollment of the index case. We did however observe a two-fold higher prevalence of DENV-reactive IgM, suggestive of recent DENV infection, in case clusters compared with control clusters. Overall, these results are broadly consistent with clustering of dengue risk around households, but indicate that any clustering is at a short temporal scale rather than sustained chains of localized transmission. These findings are relevant when considering the most effective way to deliver dengue control interventions in resource-limited endemic settings, and for understanding the spatial distribution of dengue risk in different settings.

The ‘index-cluster’ design has been used in a small number of previous studies of dengue transmission. In two studies [14,15], the primary aim was to characterize early virological and immunological events in acute DENV infection, including inapparent infection. These studies, in urban settings in Indonesia and Nicaragua, reported similar DENV infection incidence of 2–3% during a two week follow-up period, compared with ~6% in our study. The prevalence of ‘pre-enrollment’ DENV infection defined by IgM seropositivity at enrollment was 4.3% in the Nicaraguan study and 21% in the Indonesian study. Our estimate of recent DENV infection prevalence of ~12% within case clusters falls between those two reports, and in control clusters is less than half that (5%). The Indonesian and Nicaraguan studies included either no or very few control clusters, respectively, and so were not designed specifically to answer the questions about fine-scale spatial clustering of DENV infection that are the focus of our study.

An index-cluster study including controls, conducted in rural Thailand in 2004–5, found that DENV infections were highly clustered, with evidence of DENV infection in 16% of children enrolled in DENV-positive clusters and only 1% of children in negative clusters [12,13]. The authors grouped recent infections at baseline and incident infections during the two-week follow up together as one endpoint for analysis, but for comparison with our study and others, the prevalence of DENV-IgM at baseline and the two-week incidence of DENV infection can be calculated for positive clusters in the Thai study, at 5.1% and 9.1% respectively. The higher two-week incidence relative to baseline IgM prevalence suggests focal transmission associated with a very recent DENV introduction, in contrast to our study where clustered transmission appears already to have happened prior to commencement of the cluster investigation.

Differences in the study populations may have contributed to these dissimilar findings. Dengue index cases in the Thai study were detected by school absence, which is likely to be a more sensitive and timely indicator of DENV transmission than hospital admissions, as used in our study. Furthermore, the Thai study included only children whereas we enrolled both children and adults in our clusters. Adults are more likely to be partially or fully immune to DENV at baseline, and therefore a lower risk of both recent and incident DENV infections would be expected in adults than children. Surprisingly, we found the prevalence of recent infection and incidence of inapparent infection to be comparable across all age groups. The small number of symptomatic dengue cases occurred both in children <15 years and young adults 15–35 years, but not in older adults. This may be due to chance, given the small number of symptomatic cases, or may reflect partial immunity in older adults that is protective against illness but not infection. We did not detect viremia in any participants with inapparent DENV infection. Although clinically inapparent viremia has been reported [20], its relative importance to DENV transmission at a community level is not yet clear.

The fact that we observed some evidence for clustering of recent DENV infections but not incident infections around a hospitalized dengue case suggests an important temporal dimension to focal dengue transmission. Household clustering of dengue epidemiological and entomological risk justifies a focus on vector control also at the household level, which is logical since *Ae*. *aegypti* generally bite, rest and breed in and around domestic dwellings [2]. However this is sometimes extended to an assumption that vector control is best delivered by responsive targeting of residences close to reported dengue cases. This is where the temporal dimension becomes critically important. Our results show, at least in this setting, that by the time hospitalized cases are reported and a response initiated, there is no excess of incident DENV infections to be prevented. We also found no difference in DENV infection prevalence among *Ae*. *aegypti* collected from case versus control houses, although vector density in the index house at baseline was associated with a significantly higher incidence of infection within dengue positive clusters. It is possible that by limiting our follow-up period to two weeks we missed any longer chains of focal transmission, however we would expect to see some clustering signal also in our prospectively detected infections if that were the case. It is also conceivable that vector control interventions applied after illness onset in the index cases/controls could have reduced DENV infection incidence over the follow-up period, accounting for the observed lack of clustering. However we think this is unlikely to explain our findings because the lack of association persisted after adjustment for insecticide use during the 2-week follow-up period. The observation that average time spent at home was negatively associated with recent infection prevalence at baseline was unexpected and is difficult to explain, but supports the conclusion that dengue risk is not strongly clustered around households in this setting.

The strengths of this study are the large size, inclusion of both children and adults, detection of both symptomatic and inapparent DENV infections among cluster participants, and the blinding of field staff to the infection status of the index cases, thereby preventing observer bias. There are however some limitations: 1) retrospective diagnosis of index cases meant that we could not spatially and temporally match cases and controls, however we show that their distributions were similar; 2) a relatively small number of control clusters was included compared to dengue positive clusters; 3) children and young adults were under-represented among cluster participants, because they were more likely to be out at the time of the household visit and because of parental aversion to blood collection from children; 4) the use of capillary blood sampling limited the volume available for repeat diagnostic testing; 5) we may have underestimated the proportion of symptomatic infections by relying on participants’ self-reports during the intervening week between study visits, but this was not a primary endpoint of the study.

Taken together with the previous studies described above, our findings demonstrate that patterns of fine-scale spatiotemporal clustering of DENV infections differ between epidemiological settings. Marked differences in population density, human mobility and vector ecology between a highly urban setting like HCMC and the village setting of the Kamphaeng Phet index-cluster study are likely to underlie these differences. In particular we highlight differences in the temporal dimension of focal dengue transmission which suggest that in some settings like urban HCMC, reactive, and inevitably delayed, perifocal insecticide spraying around houses of reported dengue cases might not be cost-effective. Novel approaches to dengue prevention and control—such as dengue vaccines [3], antiviral chemoprophylaxis [21], and microbial control of mosquito DENV infection [22]—are likely to become available over the next decade but optimal and evidence-based application of current vector control tools will remain important, since a reduced vector density lowers the threshold for success of these novel interventions.

## Supporting Information

S1 Table Laboratory diagnostic results for index cases. (DOCX)

S2 Table DENV infections among case and control cluster participants, stratified by age. (DOCX)

S1 Fig Laboratory diagnostic algorithm for cluster participants. The flow chart shows the sequence of laboratory diagnostic assays performed on dried blood spot (DBS) or plasma samples from cluster participants, and the resulting diagnostic classifications. Incident DENV infections determined by IgM seroconversion and/or a positive NS1 or PCR result are shown in dark red. Those classified as incident DENV infections only on the basis of IgG seroconversion (a less specific definition) are shown in light red. The one participant classified as symptomatic probable dengue on the basis of IgM positivity in a single acute sample, is shown in orange. MAC/GAC-ELISA: IgM/IgG antibody capture enzyme-linked immunosorbent assay; SC: seroconvert; Pos: positive; Neg: negative; Eq-P: conversion from equivocal to positive; Atyp: atypical serology results such as positive-negative-positive across the three samples; n.d.: not done. Where noted ‘Incl 1 sympt DENV+’, this indicates that subset of participants included an acute symptomatic DENV infection.
(TIF)

S1 Checklist STROBE Checklist. (PDF)
