# Supplementary material for: Households as Foci for Dengue Transmission in Highly Urban Vietnam
Source: PLoS Negl Trop Dis. 2015 Feb 13;9(2):e0003528. doi: 10.1371/journal.pntd.0003528 (PMC4332484; doi:10.1371/journal.pntd.0003528)
Supplement: S1 Table — (DOCX) [file pntd.0003528.s001.docx]

**Table S1:** Laboratory diagnostic results for index cases

| **Laboratory diagnostic results, by index case classification** | N | (%) |
| --- | --- | --- |
| ***Dengue index cases*** | **52** |  |
| PCR-positive & NS1-positive & IgM seroconversion | 25 | 48.1 |
| PCR-positive & NS1-positive only | 7 | 13.5 |
| PCR-positive & IgM seroconversion only | 5 | 9.6 |
| NS1-positive & IgM seroconversion only | 2 | 3.8 |
| PCR-positive only | 2 | 3.8 |
| NS1-positive only | 3 | 5.8 |
| IgM seroconversion only | 5 | 9.6 |
| IgG seroconversion only^1^ | 1 | 1.9 |
| IgM positive in enrolment sample^2^ | 2 | 3.8 |
| ***Other febrile illness controls*** | **19** |  |
| Negative PCR & NS1 and no seroconversion | 19 | 100.0 |
| ***Unclassifiable*** | **6** |  |
| Negative PCR, NS1 & IgM, but IgG positive in paired samples^3^ | 3 | 50.0 |
| Negative PCR, NS1 & IgM/IgG at enrolment; no second sample | 3 | 50.0 |

^1^IgG seroconversion alone is strictly an acute flavivirus infection, not necessarily dengue. However we included this as a dengue index case because in the presence of a clinical suspicion in the HCMC setting, dengue is the most likely flavivirus.

^2^IgM seropositivity at enrolment, in the absence of PCR or NS1 positivity but presence of a clinical suspicion, is classified as presumptive dengue. For the purpose of this analysis we grouped presumptive cases with confirmed cases.

^3^Indicative of a recent flavivirus infection
