# Supplementary material for: Households as Foci for Dengue Transmission in Highly Urban Vietnam
Source: PLoS Negl Trop Dis. 2015 Feb 13;9(2):e0003528. doi: 10.1371/journal.pntd.0003528 (PMC4332484; doi:10.1371/journal.pntd.0003528)
Supplement: S2 Table — (DOCX) [file pntd.0003528.s002.docx]

|  | **Positive clusters** | | **Negative clusters** | | |
| --- | --- | --- | --- | --- | --- |
| **Cluster participants included in analysis** | **1341*** |  | **569** |  |  |
| <15 years | 291 |  | 131 |  |  |
| 15–35 years | 309 |  | 136 |  |  |
| >35 years | 735 |  | 302 |  |  |
| **Symptomatic DENV infection during 2-week follow up** | **7** | **0.52%** | **2** | **0.35%** |  |
| <15 years | 2 | 0.69% | 1 | 0.76% |  |
| 15–35 years | 5 | 1.6% | 1 | 0.74% |  |
| >35 years | 0 | 0 | 0 | 0 |  |
| **Asymptomatic DENV infection during 2-week follow up** | **75** | **5.6%** | **30** | **5.3%** |  |
| <15 years | 13 | 4.5% | 9 | 6.9% |  |
| 15–35 years | 19 | 6.1% | 7 | 5.1% |  |
| >35 years | 42 | 5.7% | 13 | 4.3% |  |
| **Recent DENV infection at baseline** | **159** | **11.9%** | **29** | **5.1%** |  |
| <15 years | 35 | 12.0% | 7 | 5.3% |  |
| 15–35 years | 36 | 11.7% | 8 | 5.9% |  |
| >35 years | 87 | 11.8% | 14 | 4.6% |  |
| **No DENV infection** | **875** | **65.3%** | **411** | **72.2%** |  |
| <15 years | 217 | 74.6% | 107 | 81.7% |  |
| 15–35 years | 196 | 63.4% | 88 | 63.8% |  |
| >35 years | 458 | 62.3% | 216 | 71.5% |  |
| **Unclassifiable** | **225** | **16.8%** | **98** | **17.2%** |  |
| <15 years | 24 | 8.3% | 7 | 5.3% |  |
| 15–35 years | 53 | 17.2% | 32 | 23.5% |  |
| >35 years | 148 | 20.1% | 59 | 19.1% |  |

**Table S2:** DENV infections among case and control cluster participants, stratified by age.
