# Supplementary figures and images for: Households as Foci for Dengue Transmission in Highly Urban Vietnam
Source: PLoS Negl Trop Dis. 2015 Feb 13;9(2):e0003528. doi: 10.1371/journal.pntd.0003528 (PMC4332484; doi:10.1371/journal.pntd.0003528)

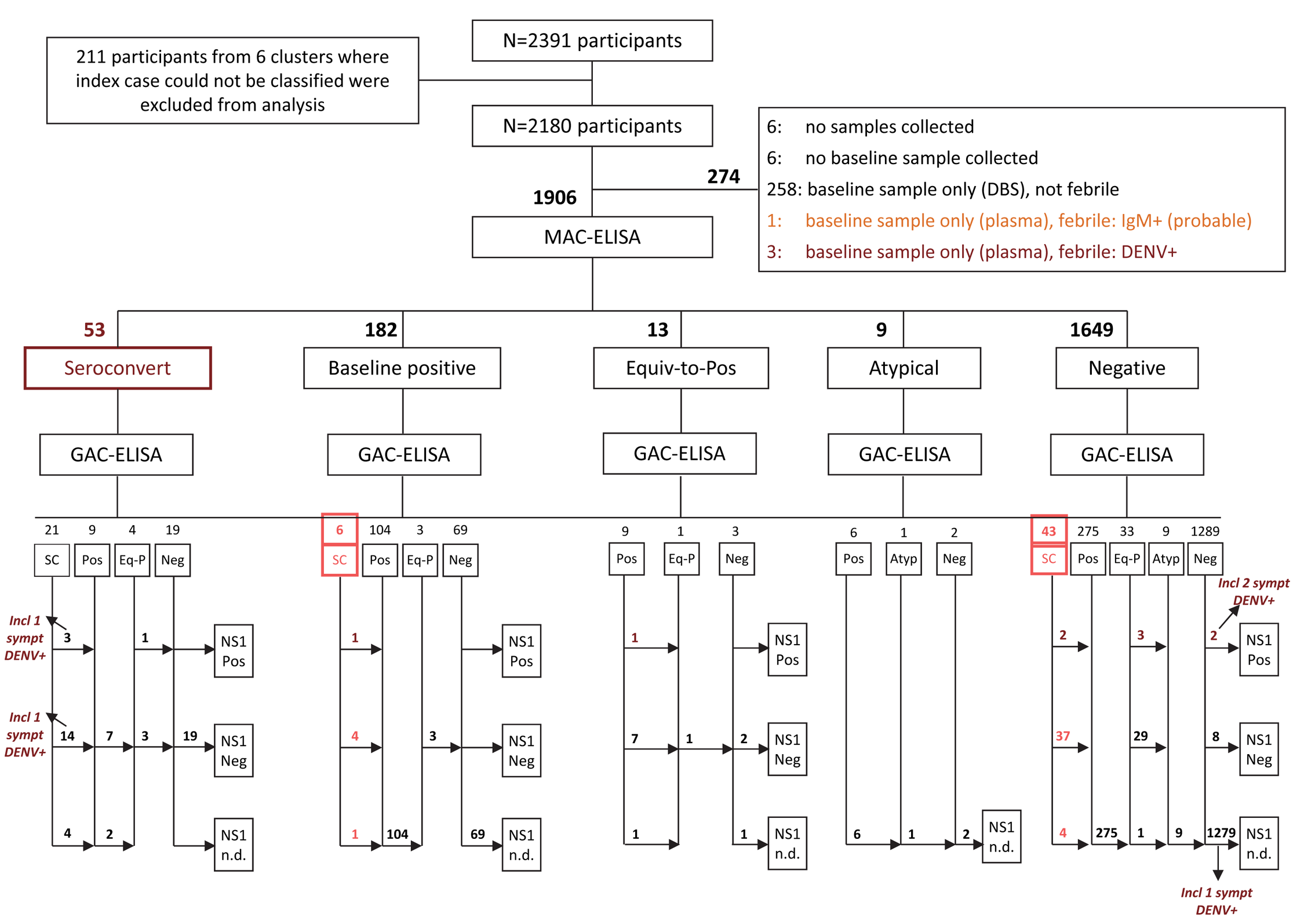

Supplement: S1 Fig — The flow chart shows the sequence of laboratory diagnostic assays performed on dried blood spot (DBS) or plasma samples from cluster participants, and the resulting diagnostic classifications. Incident DENV infections determined by IgM seroconversion and/or a positive NS1 or PCR result are shown in dark red. Those classified as incident DENV infections only on the basis of IgG seroconversion (a less specific definition) are shown in light red. The one participant classified as symptomatic probable dengue on the basis of IgM positivity in a single acute sample, is shown in orange. MAC/GAC-ELISA: IgM/IgG antibody capture enzyme-linked immunosorbent assay; SC: seroconvert; Pos: positive; Neg: negative; Eq-P: conversion from equivocal to positive; Atyp: atypical serology results such as positive-negative-positive across the three samples; n.d.: not done. Where noted ‘Incl 1 sympt DENV+’, this indicates that subset of participants included an acute symptomatic DENV infection. (TIF) [file pntd.0003528.s003.tif]
